# Vascular control of the *Drosophila* haematopoietic microenvironment by Slit/Robo signalling

**DOI:** 10.1038/ncomms11634

**Published:** 2016-05-19

**Authors:** Ismaël Morin-Poulard, Anurag Sharma, Isabelle Louradour, Nathalie Vanzo, Alain Vincent, Michèle Crozatier

**Affiliations:** 1 Centre de Biologie du Développement, UMR 5547 CNRS/Université Toulouse III and Fédération de Recherche de Biologie de Toulouse, 118 route de Narbonne 31062 Toulouse cedex 9, France

## Abstract

Self-renewal and differentiation of mammalian haematopoietic stem cells (HSCs) are controlled by a specialized microenvironment called ‘the niche'. In the bone marrow, HSCs receive signals from both the endosteal and vascular niches. The posterior signalling centre (PSC) of the larval *Drosophila* haematopoietic organ, the lymph gland, regulates blood cell differentiation under normal conditions and also plays a key role in controlling haematopoiesis under immune challenge. Here we report that the *Drosophila* vascular system also contributes to the lymph gland homoeostasis. Vascular cells produce Slit that activates Robo receptors in the PSC. Robo activation controls proliferation and clustering of PSC cells by regulating Myc, and small GTPase and DE-cadherin activity, respectively. These findings reveal that signals from the vascular system contribute to regulating the rate of blood cell differentiation via the regulation of PSC morphology.

## Abstract (web summary)

The posterior signalling centre (PSC) in *Drosophila* larva regulates blood cell differentiation but it is unclear how this is controlled. Here, the authors show that Slit/Robo signalling from the vascular system regulates PSC morphology and function, in turn, regulating blood cell differentiation.

## Graphical abstract

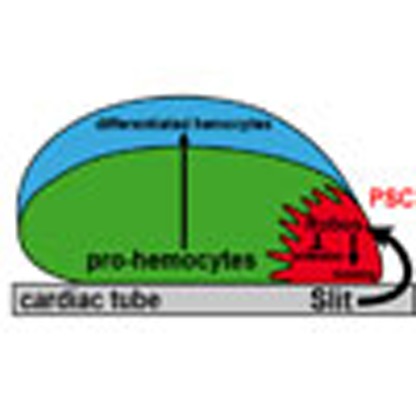

The *Drosophila* larval haematopoietic organ, called the lymph gland (LG), develops in contact with the aorta, the anterior part of the cardiac tube (CT), which corresponds to the *Drosophila* cardiovascular system. Blood cells/haemocytes differentiate in the cortex of the LG, in a so-called cortical zone (CZ), from a pool of multipotent progenitors called prohaemocytes present in the medullary zone (MZ)^1^. In addition, a group of signalling cells, termed posterior signalling centre (PSC), is clustered at the posterior end of the LG primary lobes^2^,^3^,^4^,^5^. Different signalling pathways have been shown to regulate the LG homoeostasis, that is, the balance between multipotent haemocyte progenitors and differentiated blood cells^6^,^7^,^8^,^9^,^10^,^11^,^12^,^13^,^14^,^15^. Early analyses identified key roles of the transcription factor Collier (Col)/Knot and the morphogen Hedgehog (Hh), expressed in PSC cells. Increased haemocyte differentiation in the LGs mutant for either gene suggested that the PSC plays a role equivalent to the vertebrate haematopoietic niche in the bone marrow, in controlling the balance between progenitors and differentiating cells^2^,^3^. More recent studies revealed, however, that Col expression also defined a core population of progenitors in the LG MZ, and that massive differentiation of this population occurred upon loss of Col expression in those cells^16^. The cell autonomous Col function required to maintain progenitors led to reinvestigating in more detail the PSC function under physiological conditions^16^,^17^. Data showed that, while not required for maintaining core progenitors^16^, the PSC controlled the rate of haemocyte differentiation^17^, most likely by regulating the maturation of intermediate progenitors, a heterogeneous cell population in the third instar larval LG^7^,^15^,^18^,^19^. This role of the PSC is in accordance with previous studies showing that modifying the number of PSC cells altered the LG haemocyte differentiation^3^,^6^,^14^,^20^,^21^,^22^,^23^,^24^. Reinvestigating *col* function in the LG also confirmed that the PSC plays an essential role in the mounting of a cellular immune response to wasp parasitism^2^,^5^,^16^,^17^,^25^.

We previously found that bone morphogenetic protein/decapentaplegic (BMP/Dpp) signalling in PSC cells, controlled the number of these cells, via repression of the proto-oncogene *myc*^20^. BMP signalling activity in the PSC required the expression of the Dally-like (Dlp) heparan sulfate proteoglycan (HSPG). In addition to *dlp*^20^, our unpublished transcriptome analyses of the LG in wild-type (WT) and *col* mutants identified the Robo2 receptor as being expressed in the PSC, thereby raising the question of what role Slit/Robo signalling could play in these cells.

Here we show that Slit/Robo signalling contributes to maintain the size, the morphology and the function of the *Drosophila* PSC. Robo receptors are required in PSC cells to control both the proliferation rate and the clustering of these cells. The ligand Slit is expressed in the CT, that is, the vascular system, and might signal to Robos in the PSC. On the basis of our data, we propose that inter-organ communication between the CT and the PSC is required to preserve the morphology and function of the PSC.

## Results

### Abnormal PSC morphology in *robo* mutants

**Figure 1:**
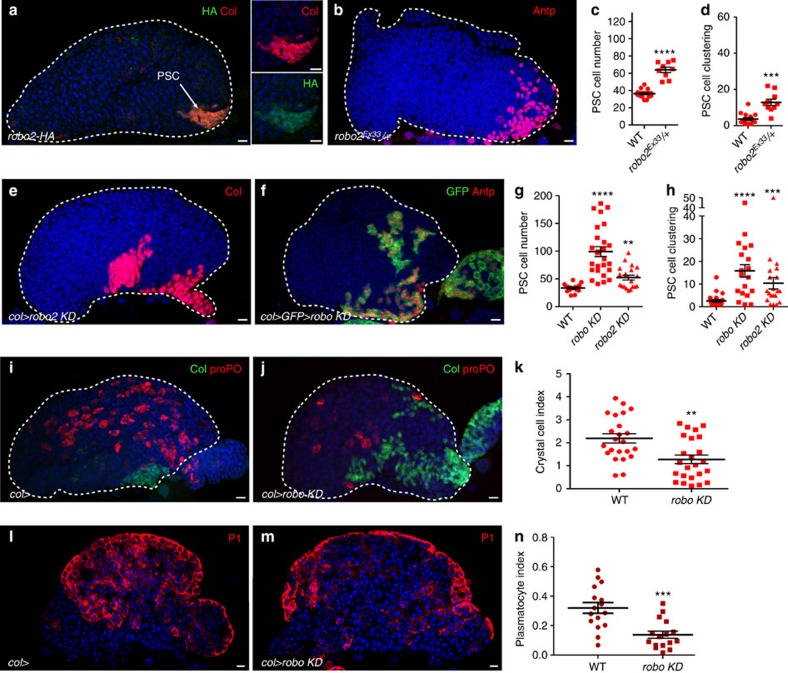
Robo2 receptor is expressed in PSC cells and Robo signalling controls PSC cell number, clustering and function. (**a**) Robo2-HA (HA, green) in the LG is expressed in PSC cells marked by Col (red). Smaller panels provide enlarged views showing co-localization of Col with Robo2-HA. (**b**) *robo2*^*Ex33*^*/+* heterozygous mutant LGs display a larger and disrupted PSC as stained by Antennapedia (Antp, red). (**e**,**f**) PSC cells are stained by Collier (col, red, **e**) or Antp (red, **f**) or express mcd8-GFP (col>GFP, green, **f**). Reducing *robo2* (*robo2 KD*) (**e**) or *robo1, 2* and *3* simultaneously (*robo KD*) (**f**) in the PSC leads to an increased number of PSC cells and their deficient clustering. (**c**,**g**) Quantification of PSC cell numbers. (**d**,**h**) Quantification of PSC cell clustering. (**i**,**j**) WT (**i**) and *robo KD* (**j**) LGs stained for PSC cells (Col, green) and crystal cells (proPO, red). Compared with WT LGs, (**i**) fewer crystal cells differentiate in *robo KD* lymph glands (**j**). (**k**) Crystal cell index. (**l**,**m**) A confocal section in the middle of the LG of WT (**l**) and *robo KD* (**m**) LGs stained for plasmatocytes (P1, red). Compared with WT LGs (**l**) fewer plasmatocytes, localized at the LG's cortex, differentiate in *robo KD* lymph glands (**m**). (**n**) Plasmatocyte index. Statistical analysis *t*-test (Mann–Whitney nonparametric test) was performed using GraphPad Prism 5 software. For all quantifications and in all figures: error bars represent s.e.m. and *P<0.1; **P<0.01; ***P<0.001; ****P<0.0001 and NS (not significant). In all figures nuclei are labelled with Topro or 4,6-diamidino-2-phenylindole (blue). Scale bars, 10 μm.

Slit/Robo signalling is a key regulator of axon guidance, cell migration, adhesion and proliferation both in vertebrates and invertebrates^26^,^27^,^28^. Three Robo receptors and one Slit, the canonical Robo ligand, are encoded in the *Drosophila* genome^26^,^29^,^30^. Examining the expression of Robo receptors by immunostaining with anti-Robo antibodies or by looking at the expression of human influenza haemagglutinin (HA)-tagged endogenous alleles^31^, showed Robo1 was detected in the MZ, the CT and at low levels in the PSC, and Robo2 in PSC cells, crystal cells and in the CT (Fig. 1a; Supplementary Fig. 1a–f). Barely detectable levels if any of Robo 3 were present in PSC cells (Supplementary Fig. 1e–f). Thus, Robo1 and Robo2 are expressed in the PSC with *robo2* at the highest level. To study the role of Robos in the LG, we first analysed a heterozygous context where one copy of robo2 was missing and observed an increase in PSC cell number (Fig. 1b,c). Furthermore, whereas PSC cells were clustered posteriorly in WT LGs (Fig. 1a; Supplementary Movie 1), the posterior clustering was lost in *robo2* heterozygous mutants (Fig. 1d). To investigate the role of *robos* in the PSC during larval development, we used a PSC-specific Gal4 driver (*col*-Gal4) to express double-stranded RNA (dsRNA) of either *robo2* alone (*robo2 KD*) or all three *robos* (termed *robo KD*). Decreased *robo2* expression in the PSC resulted in an increased PSC cell number and defects in PSC cell clustering (Fig. 1e–h). A complete rescue of PSC size and clustering was obtained when Robo2-HA was expressed in the PSC of *robo2 KD* larvae (Supplementary Fig. 1g–i), indicating that Robo2 is required in PSC cells to control their number and clustering. The ectopic expression of Robo2-HA in the PSC, the MZ or the CT did not affect the PSC cell numbers (Supplementary Fig. 2a–f,j–l). Reducing the expression of all three *robos* in the PSC (*robo KD*)-aggravated PSC cell number and clustering defects (Fig. 1f–h). Indeed, small clusters or individual PSC cells spread anteriorly along the surface of the LG (Fig. 1f; Supplementary Movie 2). A similar phenotype was observed when using *Antp*-Gal4 as another PSC driver, confirming the requirement of Robo receptors in the PSC to control its morphology (Supplementary Fig. 1j–k). Downregulating all three *robos* led to a decrease of Robo1 and 2 in PSC cells (Supplementary Fig. 1a–f) and generated a stronger PSC defect (Fig. 1f–h) compared with reducing *robo2* alone (Fig. 1e–h), indicating that another Robo, probably Robo1 could contribute to PSC morphology. This was further confirmed as only a partial rescue of PSC size and clustering was obtained when Robo2-HA was expressed in the PSC of *robo KD* larvae (Supplementary Fig. 1l–n). However, no PSC defect was observed when dsRNA against either *robo1* or *robo3* alone was expressed in PSC cells (Supplementary Fig. 1o–q). Altogether, these data indicate that *robo2* is the main Robo receptor controlling PSC morphology and that at least *robo1* might be a secondary contributor.

Controlling the PSC size was previously shown to be essential in regulating the rate of haemocyte differentiation in the LG^3^,^6^,^20^,^32^. We therefore looked at haemocyte differentiation in *col>robo KD* conditions. Crystal cells and plasmatocytes, which are the two types of differentiated haemocytes found under normal conditions, were identified by proPO and P1 antibody staining, respectively. Compared with WT, fewer crystal cells and plasmatocytes were detected in *robo KD* LGs, confirming the relevance of PSC size for the normal LG homoeostasis (Fig. 1i–n). Hh expression in the PSC was shown to be required to maintain progenitors in the MZ and to block their differentiation^3^. As in WT, the Hh-green fluorescent protein (GFP)^33^ transgene, a reporter of Hh expression in the PSC, was expressed in all PSC cells in a *robo KD* context (Supplementary Fig. 3). This suggests that in *robo KD* mutants, increased PSC size leads to an increased production of Hh signal, among others, that acts non-cell autonomously to affect the LG homoeostasis^3^.

**Figure 2:**
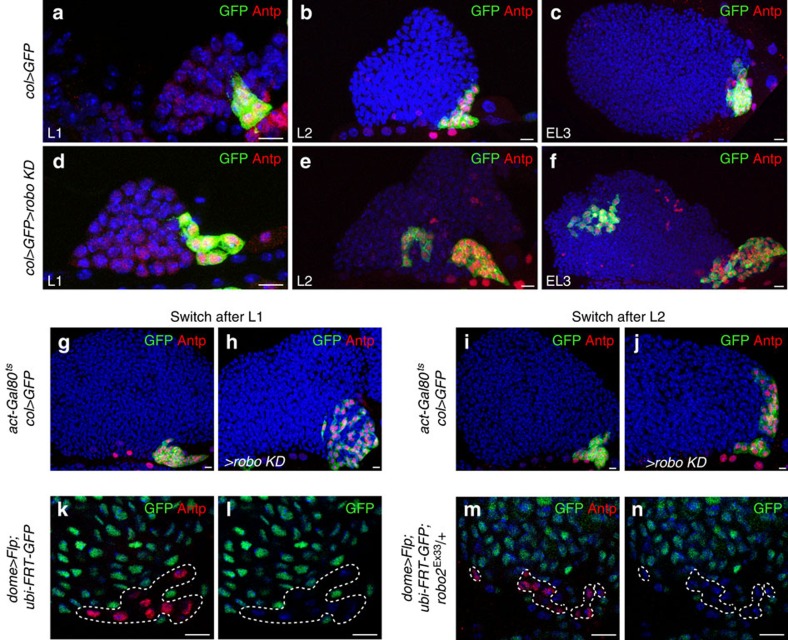
Robo receptors are required to control PSC morphology during larval development and MZ lineage tracing. (**a**–**f**) Antp (red) and GFP (green) label PSC cells in control (*col>GFP*) (**a**–**c**) and in *col>robo KD* LGs (**d**–**f**). (**a**,**d**) L1 larvae, (**b**,**e**) L2 larvae and (**c**,**f**) early L3 larvae. (**g**,**j**) Antp (red) and GFP (green) label PSC cells in Gal80ts context; control (*col>*) (**g**,**i**) and in *col>robo KD* LGs (**h**,**j**). The temperature shift (18–29 °C) was performed in L1 (**g**,**h**) or L2 (**i**,**j**), and LGs were analysed in L3. (**k**–**n**) Antp (red) labels PSC cells (**k**,**m**) and GFP (green) labels MZ lineage traced cells in control (dome>Flp; ubi>FRT>GFP) (**k**,**l**) and in *robo2*^*Ex33*^/+ heterozygous mutant (dome>Flp; ubi>FRT>GFP; robo2^Ex33^/+) (**m**,**n**). Nuclei are labelled with 4,6-diamidino-2-phenylindole (blue) (**a**–**n**). Scale bars, 10 μm.

To determine if the PSC defects observed in *robo KD* LGs are linked to PSC cell mis-specification and/or dispersion, we examined the morphology of the PSC at different larval stages. The PSC morphology defects observed in *robo KD* were not found in L1 larvae, but were detected in L2 larvae and amplified in L3, suggesting that Robos are required from the L2 larval stage (Fig. 2a–f). To go one step further, we used the temporal and regional gene expression targeting system (TARGET; Gal80ts/Gal4 expression system)^34^ to reduce *robos* in PSC cells at different time points during larval development. Loss of *robos* at the end of L1/beginning of L2 stage led to PSC morphology defects, indicating the requirement for *robos* from the L2 stage (Fig. 2g–j). To verify that scattered *robo KD* PSC cells are indeed derived from the PSC and are not *de novo* respecified MZ cells, we turned to lineage tracing to mark all cells issued from the MZ, using the Gal4 technique for real-time and clonal expression (G-TRACE) method with the *dome-Gal4* MZ-specific driver^2^,^35^. We found no PSC cells, as labelled by Antp, expressing GFP in either WT or *robo2* mutants (Fig. 2k–n). This established that the scattered PSC cells observed in *robo2* mutant LGs all originate from the PSC lineage. Altogether, these data indicate that during larval development *robo KD* PSC cells concomitantly over-proliferate and disperse.

### Slit from the cardiac tube controls PSC morphology

**Figure 3:**
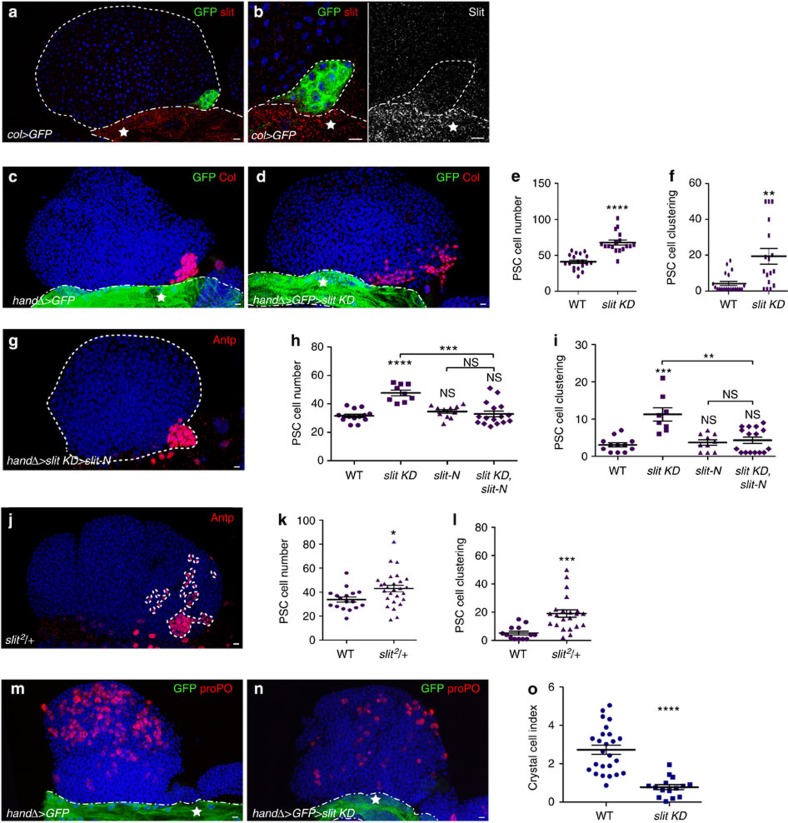
Slit expressed in the cardiac tube is required to control PSC morphology. (**a**,**b**) PSC cells express GFP (*col>GFP*, green) under the PSC *col* driver. Slit (red in **a** and **b** and white in smaller right **b**) is expressed at high levels in the cardiac tube indicated by a star. (**b**) Enlarged views showing weak Slit expression in PSC cells (red and white left and right panels, respectively). (**c**,**d**) PSC cells are labelled by Col (red) and the cardiac tube expresses GFP under the control of the cardiac tube driver handΔ (*hand*Δ*>GFP*, green). Compared with control (**c**), reducing Slit levels in the cardiac tube leads to an increased number of PSC cells and the loss of their clustering (**d**). (**e**,**f**) Quantification of PSC cell numbers and PSC cell clustering, respectively. (**g**) Antp (red) labels PSC in *hand*Δ*>slit KD>Slit-N.* The *slit KD* PSC defect is rescued by *Slit-N* overexpression in the CT. (**h**,**i**) Quantification of PSC cell numbers (**h**) and PSC cell clustering (**i**); * corresponds to the comparison with WT, whereas * above a bar indicates the two conditions being compared. (**j**) Antp (red) labels the PSC in *slit*^*2*^*/+* heterozygote mutant. (**k**,**l**) Quantification of PSC cell numbers (**k**) and PSC cell clustering (**l**). (**m**,**n**) Crystal cells are labelled by prophenoloxidase (proPO) antibody (red) and the cardiac tube expresses GFP (*hand*Δ*>GFP*, green) under the control of the cardiac tube driver *Hand*Δ. Fewer crystal cells differentiate in LGs, when *slit* expression is decreased in the cardiac tube (**n**), compared with WT (**m**). (**o**) Crystal cell index in **m** and **n** contexts. Statistical analysis *t*-test (Mann–Whitney nonparametric test) was performed using GraphPad Prism 5 software. Scale bars, 10 μm.

Slit is the canonical ligand of *Drosophila* Robos. High levels of Slit were detected in the CT and low levels in PSC cells (Fig. 3a,b). Expression of *slit* dsRNA in the CT using the handΔ-Gal4 driver, resulted in barely detectable Slit levels in both the CT and the LG (Supplementary Fig. 4c–d), indicating that the source of Slit is the CT and that Slit in the PSC has diffused from the CT. In support of this conclusion, reducing *slit* expression (*col>slit KD*) in PSC cells (Supplementary Fig. 4g–h) had no effect on PSC morphology, while reduction of *slit* expression specifically in the CT using either handΔ-gal4 or another CT driver NP1029-gal4 (ref. 36), led to both increased number and defective clustering of PSC cells (Fig. 3c–f; Supplementary Fig. 4e–f). PSC morphology defects observed in *hand*Δ*>slit* KD LGs were rescued by co-expressing an active form of Slit (Slit-N; Fig. 3g–i; ref. 37), further confirming that Slit from the CT is required to control PSC morphology. A similar PSC phenotype was observed when one copy of *slit* was missing (Fig. 3j–l). *Slit KD* experiments were performed in larvae once the CT had formed to avoid any CT morphological defects due to Slit/Robo signalling requirement for embryonic CT development^38^,^39^,^40^,^41^,^42^ (Supplementary Fig. 4g–h). To determine whether an ectopic source of Slit might affect the PSC, we expressed Slit-N in the PSC, the MZ or the CT cells. No effect on PSC morphology was observed, indicating that Slit from the CT is sufficient to activate Robo signalling in PSC cells (Supplementary Fig. 2g–l). Consistent with the increased PSC cell number observed in *slit KD* LGs, fewer crystal cells were present in the LGs when *slit* expression was downregulated in the CT (Fig. 3m–o). Altogether, these data indicate that downregulating *slit* in the CT caused a phenotype similar to downregulating *robos* in the PSC. These data strongly suggest that Slit/Robo signalling mediates communication between the vascular system and the PSC to control PSC cell number, their clustering and ultimately their function.

### Robos control the accumulation of Dally like in the PSC

**Figure 4:**
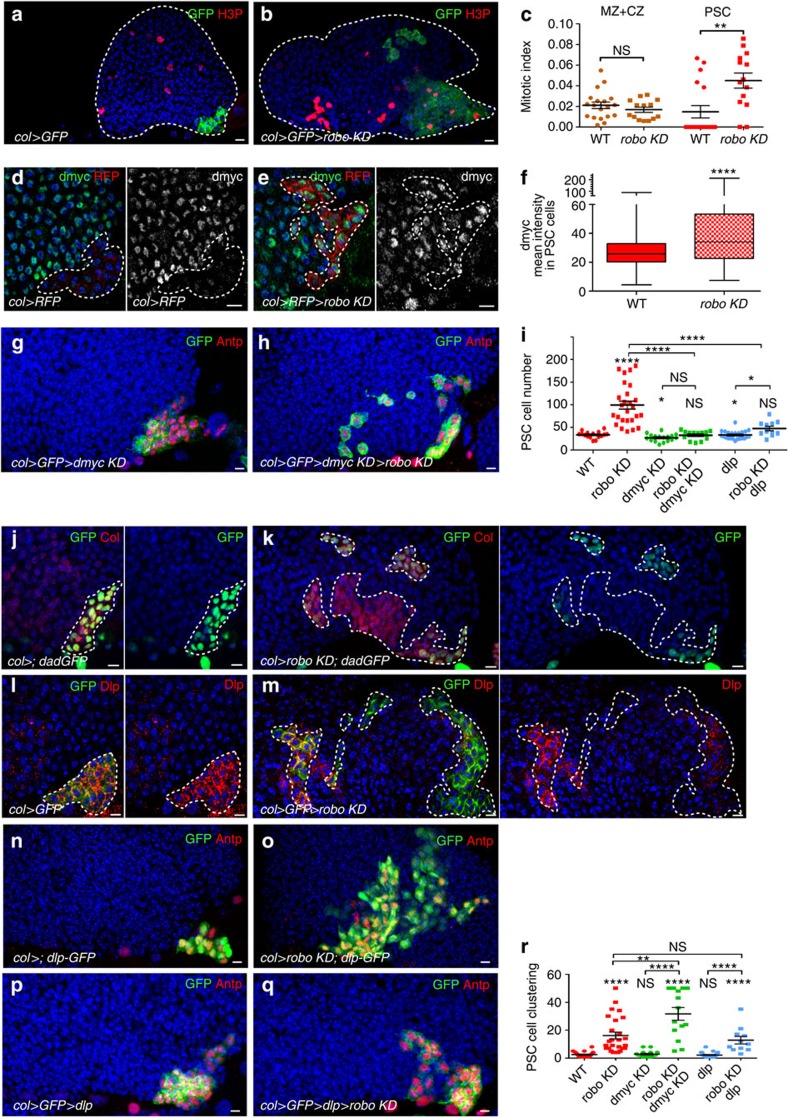
Robo receptors in the PSC control PSC cell proliferation through the activation of the BMP/Dpp signalling pathway. (**a**,**b**) Phosphor-histone H3 (H3P) staining (red) in the presence or absence of *robos* in PSC cells (green). (**c**) Mitotic index in the (MZ+CZ), and PSC in WT and *robo KD* LG. (**d**–**e**) PSC cells express RFP (red) under the PSC *col* driver in WT (**d**) and in *robo KD* (**e**). dmyc staining (green, small left panels and white small right panels. (**f**) Quantification of dmyc mean intensity in PSC cells in WT and in *robo KD*. (**g**,**h**) PSC cells are stained by Antp (red) and express GFP (Col>GFP, green). Reduction of *dmyc* expression in the PSC (**g**) slightly reduces the number of PSC cells; *dmyc* reduction in the *robo KD* PSC rescues the number of PSC cells, but not their clustering (**h**). (**i**,**r**) Quantification of PSC cell numbers (**i**) and PSC cell clustering (**r**) under various mutant conditions. (**j**,**k**) Daughters against dpp-GFP (dad-GFP, green) are expressed in PSC cells labelled by Col (red). Compared with WT (**j**) *robo KD* PSC (**k**) expresses a lower level of dad-GFP. (**l**,**m**) Dally-like (dlp, red) is expressed in PSC cells labelled by GFP (green). Compared with control (**l**), *robo KD* PSC (**m**) expresses a lower level of Dlp. (**n**,**o**) PSC cells express GFP (green) and are stained by Antp (red). (**p**,**q**) Antp (red) and Dlp-GFP (green) label PSC cells in WT (**p**) and *robo KD* (**q**). All PSC cells express GFP both in WT and *robo KD*. The overexpression of Dlp in the PSC slightly reduces the number of PSC cells (**i**,**r**); in *robo KD*, this restores a WT number of PSC cells, but not their clustering (**i**,**p**–**r**). Statistical analysis *t*-test (Mann–Whitney nonparametric test) is performed using GraphPad Prism 5 software. Scale bars, 10 μm.

To determine whether cell proliferation was affected in *robo KD* LGs, we analysed the expression of the phospho-histone H3 (H3P) M-phase marker and calculated the mitotic index in the PSC and in the MZ+CZ (Fig. 4a–c). In agreement with the increased PSC size, the number of mitotic cells was statistically higher in the PSC of *robo KD* larvae than in WT. No change in the mitotic index in the other LG cells was found, indicating that Robo receptors specifically modulate cell proliferation in the PSC without affecting that of other LG cells. Since proliferation of PSC cells is controlled by expression levels of the proto-oncogene *dmyc*^20^,^32^, we questioned whether increased proliferation in the *robo KD* PSC was linked to *dmyc* misregulation. Indeed in *robo KD* PSC, *dmyc* expression in the PSC is higher than in WT (Fig. 4d–f). We therefore asked whether decreasing *dmyc* could rescue *robo KD* PSC defects. Simultaneous reduction of *dmyc* and *robo* (*col*>*robo KD*>*dmyc KD*; Fig. 4g–i) restored WT PSC cell number, but did not rescue clustering defects (Fig. 4r). To determine whether rescue of the PSC cell number resulted from either cell death or reduced proliferation, we immune-stained LGs for the apoptotic marker Dcp-1 (ref. 43) and the mitotic marker H3P. While we could not observe Dcp-1 staining in PSC cells in any condition (Supplementary Fig. 5 a–d), indicating the absence of cell death, we found that the PSC mitotic index was significantly lower in rescued larvae *(col>robo KD>dmyc KD)* as compared with *robo KD* larvae (Supplementary Fig. 5e). Altogether, these data established that *robos* control PSC cell proliferation via *dmyc* and clustering by other mechanisms. *dmyc* transcription in the PSC is under positive and negative regulation by Wnt/Wg and BMP/Dpp signalling, respectively^20^. We therefore examined whether Wnt/Wg signalling was affected. D-frizzled 3 (dfz3) is a target of the Wnt/Wg pathway^44^, and the transgene dfz3-red fluorescent protein is a reporter of the Wnt/Wg pathway activity in the PSC. Reduction of *robo* function in the PSC did not affect dfz3-RFP expression, indicating that Wnt/Wg signalling was not impaired (Supplementary Fig. 6). Furthermore, to determine whether BMP/Dpp signalling was affected, we analysed the expression of dad-GFP, a reporter of the pathway^20^. Reduction of *robo* function in the PSC led to a decrease in dad-GFP expression. This effect, however, was not uniform but varied from cell to cell independently of their position (Fig. 4j,k; Supplementary Fig. 7a). This stochastic reduction of dad-GFP expression indicates that Robo signalling is required for robust BMP activity in all PSC cells. Activation of BMP/Dpp signalling requires the Dlp HSPG in PSC cells^20^. A strong decrease in the Dlp level was observed in *robo KD* PSC (Fig. 4l,m; Supplementary Fig. 7b). To define whether Robos regulate *dlp* at the transcriptional or protein levels, we analysed both the expression of a GFP reporter under the control of the *dlp* promoter (dlp-GFP) and *dlp* by quantitative PCR with reverse transcription. In *robo KD* LGs, *dlp-GFP* expression is similar to WT (Fig. 4n,o) and no change in *dlp* RNA levels could be detected (Supplementary Fig. 7c). This suggests that Robo signalling does not control *dlp* transcription, but rather Dlp protein level in PSC cells. We then asked whether restoring *dlp* could rescue *robo KD* PSC defects. Overexpression of *dlp* in *robo KD* PSC (col>*robo KD*>*dlp*) did indeed result in a normal number of PSC cells, but did not rescue their clustering (Fig. 4i,p–r), indicating that Robos regulate PSC cell proliferation by controlling Dlp accumulation.

### DE-cadherin controls PSC cell numbers and their clustering

**Figure 5:**
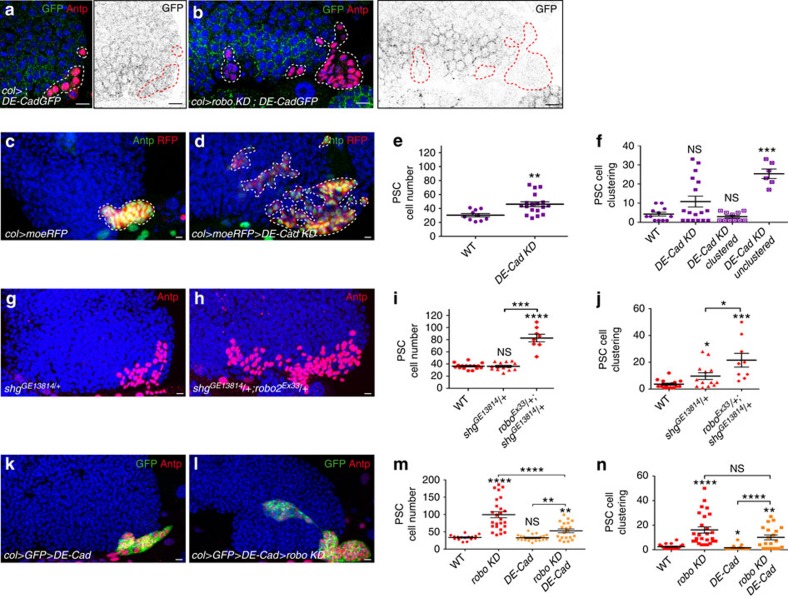
Robo receptors control the number and clustering of PSC cells via DE-cadherin. (**a**,**b**) L2/early L3 LGs where DE-cadherin-GFP (green in left panels; black in right panels) is expressed in PSC cells labelled by Antp (red). Compared with WT (**a**) *robo KD* LGs (**b**) express a lower level of DE-cadherin-GFP in the PSC. (**c**,**d**) Antp (green) and RFP (*col>moeRFP*, red) label PSC cells in control (**c**) and in *col>DE-cadherin KD* PSC (**d**). (**e**,**f**,**i**,**j**,**m**,**n**) Quantification of PSC cell numbers (**e**,**i**,**m**) and PSC cell clustering (**f**,**j**,**n**) in various mutant conditions. Reduction of *DE-cadherin* in the PSC (**d**) leads to an increased number of PSC cells compared with control (**c**). Averaging the clustering of all *DE-Cad KD* LGs (*n*=44 lobes) does not reveal a significant clustering defect. However, these LGs can be divided into two classes: clustered (66%) and unclustered (33%), revealing a clustering defect (**f**). (**g**,**h**) Antp (red) labels the PSC in *DE-cadherin* mutant (*shotgun, shg*) *shg*^*GE13814*^*/+* heterozygote (**g**) and *shg*^*GE13814*^*/+*; *robo2*^*EX33*^*/+ trans*-heterozygote mutant (**h**). A stronger PSC defect is observed in *shg*^*GE13814*^*/+*; *robo2*^*EX33*^*/+ trans*-heterozygote compared with the single *shg*^*GE13814*^*/+* heterozygote mutant. For robo2^Ex33^/+ *trans*-heterozygote mutant, analysed in parallel to *shg*^*GE13814*^*/+*; *robo2*^*EX33*^*/+ trans*-heterozygote, the quantification of PSC cell numbers and clustering for *robo2*^*Ex33*^*/+* heterozygote is given in Fig. 1c,d. (**k**,**l**) Antp (red) and GFP (green) label PSC cells when DE-cadherin (DE-Cad) is overexpressed (**k**) or when *robo KD* and DE-cadherin are co-expressed in the PSC (**l**). Statistical analysis *t*-test (Mann–Whitney nonparametric test) is performed using GraphPad Prism 5 software.

The next step was to address how Robos control PSC cell clustering. Robo signalling inhibits cadherin-mediated adhesion in various cell types both in vertebrates and in *Drosophila*^38^,^40^,^45^. *Drosophila* epithelial (DE)-cadherin has been reported to be expressed in the MZ of L3 larval LGs^3^,^46^. To examine its expression in the PSC during larval development, we used a GFP-tagged DE-cadherin (DE-CadGFP) expressed under its endogenous promoter^47^. In WT LGs, DE-CadGFP was detected in the PSC at the L2 stage. Barely, detectable levels were observed in L2 *robo KD* PSCs, compared with WT, indicating that Robos control DE-CadGFP accumulation in PSC cells (Fig. 5a,b). To test whether DE-cadherin is required for PSC cell clustering, we expressed *DE-cadherin* dsRNA in PSC cells. We observed that 33% of the LGs (*n*=44 lobes) exhibited a defect in PSC cell clustering (Fig. 5c–f). Unexpectedly, an increased number of PSC cells was also found (Fig. 5c–e), indicating that DE-cadherin is required to control both PSC cell number and clustering. We then asked whether the increased proliferation of PSC cells observed upon DE-cadherin removal from the PSC was linked to impairment of BMP/Dpp signalling. For this we examined dad-GFP expression and Dlp accumulation (Supplementary Fig. 8). Reduced DE-cadherin in the PSC led to decreased dad-GFP and Dlp expression, suggesting that DE-cadherin is required for normal accumulation of Dlp protein in PSC cells and in turn BMP activity, and ultimately PSC cell number. *DE-cadherin* (*shg*) heterozygous mutants showed a mild PSC phenotype that was increased in *robo2* and *DE-cadherin trans*-heterozygous mutant LGs, confirming that DE-cadherin and Robo2 act together to control PSC morphology (Fig. 5g–j). Furthermore, while PSC-specific expression of DE-cadherin had no effect by itself, its expression in *robo KD* partially rescued PSC cell numbers (Fig. 5k–m). Increasing DE-cadherin expression in *robo KD* PSC cells does not rescue the PSC cell clustering defect (Fig. 5n). However, the method used to quantify PSC clustering reflects PSC cell dispersion, but does not take into account the number of PSC cell clusters. Counting PSC cell clusters indicated that while an average of 27 clusters (*n*=26 lobes) was measured in *robo KD*, ∼5 (*n*=20 lobes) were observed when DE-cadherin was overexpressed in *robo KD* LGs. This indicates that PSC overexpression of DE-cadherin in *robo KD* results in the formation of larger PSC cell clusters compared with *robo KD* alone. In conclusion, Robos are necessary for the accumulation of DE-cadherin in PSC cells, which in turn controls both their numbers and clustering.

### Robos control PSC cell clustering by repressing Cdc42 activity

**Figure 6:**
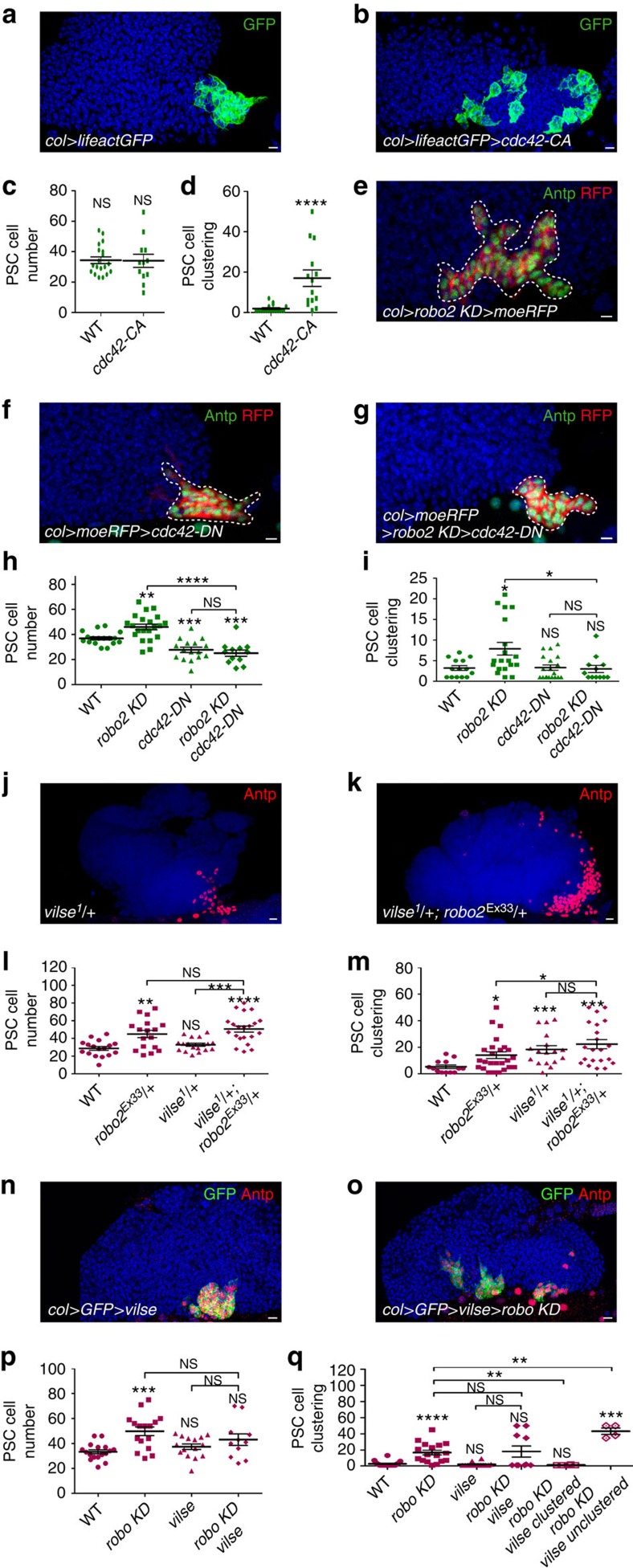
Robo receptors control PSC cell clustering by repressing Cdc42 activity. (**a**,**b**) LifeactGFP (green) is expressed in PSC cells under the control of the col driver (*col>*) (**a**) and co-expressed with a constitutive form of Cdc42 (cdc42-CA) (**b**). (**c**,**d**) Quantification of PSC cell numbers (**c**) and PSC cell clustering (**d).** (**e**–**g**) Antp (green) and RFP (col>moeRFP, red) label PSC cells in *robo2 KD* (**e**), when a dominant negative form of Cdc42 (*cdc42-DN*) is expressed (**f**) or when *robo2 KD* and *cdc42-DN* are co-expressed in the PSC (**g**). While the expression of *cdc42-DN* has no major effect on PSC cells (**f**), its co-expression in *robo2 KD* (**g**) rescues the *robo2 KD* PSC defect (**e**). (**h**,**i**) Quantification of PSC cell numbers (**h**) and PSC cell clustering (**i**). (**j**,**k**) Antp (red) labels PSC cells in *vilse*^*1*^*/+* heterozygous mutant (**j**) and in *vilse^1^/+*; *robo2*^*Ex33*^*/+ trans*-heterozygous mutant (**k**). (**l**,**m**) Quantification of PSC cell number (**l**) and PSC cell clustering (**m**). A PSC cell clustering defect is observed in vilse^1^/+ heterozygous mutant. (**n**,**o**) Antp (red) labels PSC cells in *col>vilse* (**n**) and *col>vilse>robo KD* (**o**). (**p**,**q**) Quantification of PSC cell numbers (**p**) and PSC cell clustering (**q**). Averaging the clustering of all *col>robo KD>vilse*, LGs does not reveal a significant clustering defect (Fig. 6q). However, these LGs (*n*=10 lobes) can be divided into two classes: clustered (60%) and unclustered (40%), revealing a partial rescue of PSC cell clustering defect. Statistical analysis *t*-test (Mann–Whitney nonparametric test) was performed using GraphPad Prism 5 software. Nuclei are labelled with Topro (blue). Scale bars, 10 μm.

Incomplete rescue of the *robo KD* PSC clustering phenotype by DE-cadherin overexpression indicated that other Robo targets are involved. The small RhoGTPase Cdc42, which acts on actin dynamics, was one obvious candidate, since many studies have shown that Robo signalling represses Cdc42 activity^30^,^48^. While the expression of a constitutively active form of Cdc42 (Cdc42-CA) led to a clustering defect without affecting PSC cell number (Fig. 6a–d), the expression of a dominant negative form of Cdc42 (Cdc42-DN) in the PSC did not affect clustering, but slightly decreased the number of PSC cells (Fig. 6f,h,i). These data indicate that Cdc42 must be inactivated to maintain PSC cell clustering. To determine whether Cdc42 inactivation and Robo signalling are functionally linked, we performed rescue experiments. The expression of the inactive form (Cdc42-DN) in *robo2 KD* PSC rescued cell clustering defects (Fig. 6e–i), showing that Robo signalling controls PSC cell clustering by repressing Cdc42 activity.

*Vilse* encodes a Rho GAP that binds a conserved domain (CC2) in the intracellular part of Robo1, and links Robo1 signalling to Rac and Cdc42 activities in *Drosophila* tracheal cells and axons^49^,^50^. However, Vilse does not bind the Robo2 receptor^34^,^49^. We analysed the PSC morphology in *vilse*^*1*^*/+* heterozygous mutant LGs. While no changes in PSC cell number were observed, there was a clustering defect (Fig. 6j–m), suggesting that *vilse* could contribute to PSC cell clustering. To test this possibility, we performed rescue experiments by expressing *vilse* in *robo KD* PSCs. We observed that 60% of the LGs (*n*=10 lobes) did not have clustering defects, indicating a partial rescue of the PSC cell clustering defect (Fig. 6n–q). Altogether, this suggests that *vilse* might function downstream of Robo1 to control PSC cell clustering via the regulation of Cdc42 activity, and that Robo1 and Robo2 contribute to the cohesiveness of the PSC.

## Discussion

**Figure 7:**
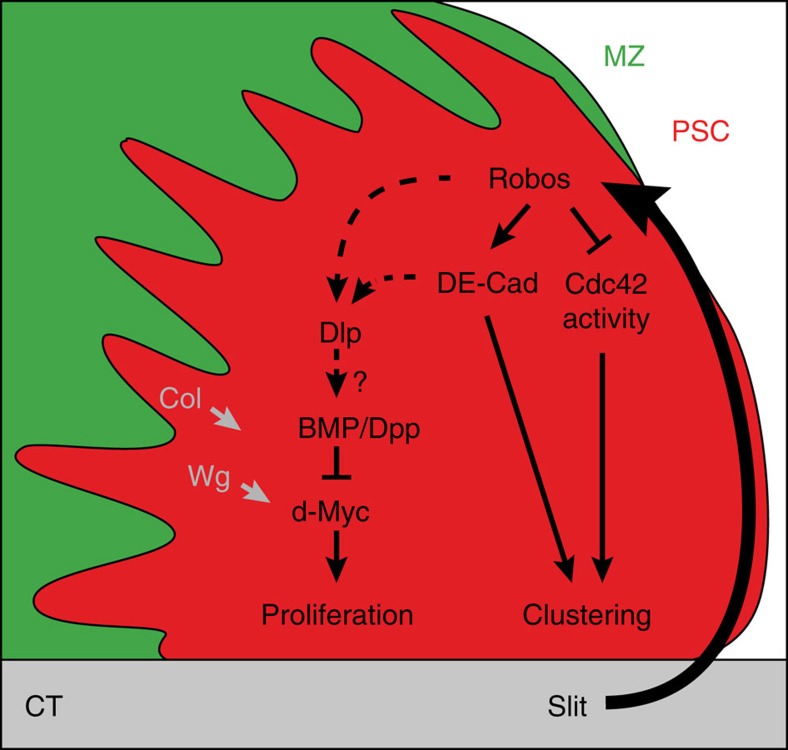
An integrative model for the role of Robo signalling in the PSC. Our schematic diagram of a WT LG with the PSC in red, the MZ in green and the CT in grey. Slit from the CT activates the Robo signalling pathway in the PSC. Robo signalling controls PSC cell clustering and proliferation. Proposed interactions between Robos, bone morphogenetic protein/decapentaplegic (BMP/Dpp) signalling, DE-cadherin and Cdc42 activity for the control of PSC cell proliferation and clustering are indicated. Dlp expression in the PSC is also controlled by Collier/Knot (Col/kn) transcription factor, and Wint/Wingless (Wnt/Wg) signalling activates *dmyc* expression^20^.

The dependence of haematopoietic cell homoeostasis on signals from the niche has been established in both vertebrates and *Drosophila*^2^,^3^,^51^,^52^,^53^,^54^. *Drosophila* PSC size must be controlled to maintain normal LG haemocyte differentiation. However, the mechanisms that control the size and the morphology of niches are poorly understood. Here we provide evidence that the *Drosophila* CT is required to maintain the PSC morphology and in turn its function. This is the first study that reports on communication between the vascular system and the PSC in *Drosophila*. We propose that this communication is mediated by Slit/Robo signalling. Slit from the CT activates Robo receptors in the PSC, which control both the number and clustering of PSC cells. Our data reveal a new signalling cascade, with Robo acting on the accumulation of the HSPG Dlp, the activation of BMP/Dpp signalling in the PSC and controlling PSC cell proliferation via *dmyc* repression. Furthermore, our data establish that Robos also act via DE-cadherin upregulation and Cdc42 inactivation in order to modulate PSC cell clustering. An integrative model is given in Fig. 7.

Since its discovery as a key regulator of axon guidance, both in *Drosophila* and in vertebrates, the Slit/Robo signalling pathway has been implicated in the regulation of different developmental processes, including cell adhesion, cell migration and cell proliferation, depending upon the tissue context. It has also been shown to act either as an oncogene or as a tumour suppressor^26^,^28^,^55^. Recent studies have established that in addition to the canonical Slit/Robo signalling pathway, Slit is a ligand for Dscam1 in a Robo independent pathway^56^. Our finding that Slit and Robos both regulate proliferation and clustering of *Drosophila* PSC cells, strongly suggests that canonical Slit/Robo signalling is active in PSC cells and coordinates both processes in the same group of cells. An independent study reported that *bag of marbles* (*bam*), a putative translational regulator, controls the number and clustering of PSC cells, through its interactions with the Insulin-like growth factor pathway and the Retinoblastoma (Rbf)-family protein^32^. It was proposed that Bam/Rbf could regulate PSC cell numbers by repressing *dmyc* expression in parallel to BMP(Dpp)/Wnt(Wg) signalling, but the PSC clustering defect was not addressed. Thus, several signalling pathways may converge to fine-tune the number and clustering of PSC cells. Whether Robo signalling and Bam/Rbf interact to control PSC cell clustering remains an open question.

Previous studies established that Slit/Robo signalling regulates cell motility or adhesion by controlling the activity of small Rho GTPases such as Rho, Rac and Cdc42, as well as cadherin, but the underlying molecular mechanisms are poorly understood so far^30^,^45^,^57^. In *Drosophila* tracheal cells and axons, activation of Robo1 by Slit results in the recruitment and activation of Vilse, a Rac/Cdc42 GAP. Vilse binds to one conserved domain (called CC2) of the intracellular domain of the Robo1 receptor, which is not found in Robo2 (refs 49, 50). We have now shown that PSC cell clustering requires the inactivation of Cdc42 under the control of Slit/Robo signalling and that Vilse could contribute to this inactivation, probably by binding Robo1. Recent data reported that in *Drosophila*'s neurons Robo2 binds to and prevents Robo1 signalling^58^, and that in tendons cells Robo2, which is essential for Slit processing^37^, can bind LRT, a leucine-rich repeat protein required to target muscles to tendon cells^59^. How Robo2 and Robo1 interact in PSC cells to control PSC morphology remains to be discovered.

Proper formation of the *Drosophila* embryonic CT requires both Robo1 and Robo2 receptors, which act in part by controlling the dynamic distribution of DE-cadherin in post-mitotic cardiomyocytes during lumen formation^38^,^39^,^40^,^42^. Our data show that Robos control the expression of DE-cadherin in PSC cells and that DE-cadherin regulates PSC cell clustering and proliferation via the accumulation of the HSPG Dlp. Previous studies performed in mammary epithelial tumour cells pointed to coordinated changes in the expression of Syndecan1, another transmembrane HSPG, and E-cadherin during epithelial cell transformation^60^. Taken together, these studies and our data suggest that the control of HSPG distribution by cadherin is used both in mammals and invertebrates, and could be involved in either normal development or in tumorigenic processes.

In mammals, the microenvironment that controls HSC self-renewal and differentiation in the bone marrow has two components: an endosteal (osteoblastic) niche and a vascular niche^52^,^53^,^54^. Recent profiling studies indicated that Slit ligands (Slit1-3) and Robo receptors (Robo 1–4) are expressed in the mouse bone marrow and that Slit/Robo signalling may play a role in HSCs homoeostasis^61^,^62^,^63^. Slit2 plays a role in regulating *in vitro* osteoblast differentiation^64^. A recent study also established that Robo4 promotes the integrity of vessels in the bone marrow and regulates HSC entry^65^. In vertebrates, adult HSCs are specified from haemogenic endothelial precursors of the aorta-gonad-mesonephros^66^,^67^. In *Drosophila*, the LG blood cell progenitors and vascular cells both originate from the embryonic cardiogenic mesoderm^68^, thus highlighting evolutionary parallels with haemogenic endothelium in vertebrates. Considering the low genetic redundancy in *Drosophila*, and the high degree of conservation of fundamental cellular functions and signalling pathways between *Drosophila* and vertebrates, there is promise that our newly identified regulation will further unravel the complex process of haematopoiesis and its evolution in bilaterians.

## Methods

### Fly strains

The fly strains were as follows: w^118^(WT), UAS-mcd8GFP^2^, dad-GFP (J. Casanova, Institut de Biologia Molecular de Barcelona, Barcelona, Spain), robo2^Ex33^ (G. Bashaw, University of Pennsylvania School of Medicine, Philadelphia, UAS-Robo2-HA (B. J. Dickson, Research Institute of Molecular Pathology, Vienna, Austria), *vilse*^1^ and UAS-vilse (C. Samakovlis, Wenner-Gren Institute, Stockholm, Sweden), UAS-DE-cadherin and UAS–dmyc ds (C. Benasayag, Centre de Biologie du Développement, Université Toulouse III, Toulouse, France), DE-cadherin-GFP (Y. Hong, University of Pittsburgh School of Medicine, Pittsburgh, USA), Antp-Gal4 and *UAS-RedStinger*, *UAS-ubi-STOP-Stinger*, *UAS-Flp/CyO* (G-TRACE), (U. Banerjee, Molecular Biology Institute, University of California, Los Angeles, USA), and handΔ-Gal4 and NP 1029-Gal4 (L. Perrin, TAGC/UMR 1090, Université, Aix Marseille, France). HandΔ-Gal4 corresponds to the hand visceral (HV element) described in ref. 69. UAS-robo1, 2 and3 ds (robo KD) are described in ref. 70 and *UAS-Slit-N* (T. Volk, Weizmann Institute of Science, Israel). Dlp-GFP (D. Harrison, University of Kentucky, USA), Dfrz3-RFP (gift from Dani Osman), UAS-robo2-HA^31^, *dad-GFP, Hh-GFP* *col-Gal4* and *dome-Gal4* (ref. 2). RNA interference (RNAi) strains were provided by the Bloomington and the Vienna *Drosophila* RNAi stock centres: *islit* (VDRC 108853 and BL 31467), *irobo1* (VDRC 100624 and BL 31663), *irobo2* (VDRC 11823 and BL 34589), *irobo3* (VDRC44702 and BL 29398) and *iDE-cad* (VDRC 103962). For *islit* and *irobo2*, we mainly used VDRC 108853 and BL 34589, respectively. All other strains were provided by the Bloomington stock center. For RNAi treatments, *UAS-Dicer 2* was introduced and *Drosophila* development proceeded at 18 °C until L1 stage before shifting to 29 °C.

### Antibody staining

Staining procedures were performed as described elsewhere^19^,^20^, using mouse anti-Col (1/200; ref. 2; guinea-pig anti-Col (1/5,000; A. Moore, Doshisha University, Kyotanabe, Kyoto, Japan); rabbit anti-H3P (1/200; Upstate Biotechnology); mouse anti-proPO (1/200; T. Trenczel, Justus-Liebig-University Giessen, Giessen, Germany); anti-P1 (1/30; I. Ando, Institute of Genetics, Biological Research Center of the Hungarian Academy of Science, Szeged, Hungary); mouse anti-Antp (1/100), anti-Dlp (1/50), anti-Robo1 (1/10), anti-Robo3 (1/10) and anti-Slit (1/10; Hybridoma Bank); rabbit anti-Robo2 (1/200; B. J. Dickson, Research Institute of Molecular Pathology, Vienna, Austria); and mouse anti-HA (HA11; 1/100; Covance) and rabbit anti-Dcp-1(Asp216) (antibody #9,578, 1/200; Cell Signaling Technology).

### Quantification of PSC cell numbers

In all experiments, all genotypes were analysed in parallel and quantifications (either for PSC cell number or PSC cell clustering) given in one panel correspond to one experiment. Each experiment was repeated independently at least three times. PSC cells were counted manually using Fiji multi-point tool software. Statistical analyses *t*-test (Mann–Whitney nonparametric test) was performed using GraphPad Prism 5 software.

### Quantification of PSC cell clustering

3D Volocity software was used to define the ROIs (region of interests) corresponding to the PSC stained by Antp, GFP or RFP expressed under a PSC driver. The ‘close' function was used to increase artificially the size of the ROIs, leading to fusion of the touching areas. The number of iterations of the ‘close' function was increased until the number of ROIs per lobe reached one. The more scattered was the PSC, the more distant were the initial ROIs, and the higher was the iteration number. The number of iterations thus measures ‘PSC cell clustering'. For computing time reasons, the iteration maximum was stopped at 50 times.

### Quantification of expression intensity per cell

Three slides per stack were analysed. Fiji software was used to define the ROIs corresponding to PSC cells. The mean intensity for Hh-GFP, dad-GFP and Dlp in each ROI was quantified. For nuclear staining such as for Hh-GFP and dad-GFP, Antp labelling was used to define ROIs. For membrane staining such as for Dlp, mcd8-GFP expressed under the Pcol driver was used to define ROIs.

### Mitotic index measurement

For counting mitotic cells, anti-H3P staining was performed on Pcol>GFP (GFP-labelled PSC cells) and Pcol>GFP>robo KD LGs. The LG size fluctuates from one larva to another, even in synchronised WT larvae. Measuring the mitotic index is therefore the most reliable way to determine how proliferation is affected in a given mutant context, since it takes into account the variation in size between individual LGs of the same genotype. The mitotic index in the PSC was measured by dividing the total number of PSC cells by the number of H3P-positive cells. The mitotic index in the MZ and CZ was measured by dividing the total number of MZ and CZ cells by the number of H3P-positive cells. Using ICY software, the total number of cells stained by 4,6-diamidino-2-phenylindole in the PSC, MZ and CZ was quantified, and the number of mitotic figures in a given LG was counted. At least 14 anterior lobes were scored per genotype. Statistical analyses *t*-test (Mann–Whitney nonparametric test) was performed using GraphPad Prism 5 software).

### Crystal cell and plasmatocyte quantification

LGs were stained with proPO antibody (crystal cell) or P1 antibody (plasmatocyte) and 4,6-diamidino-2-phenylindole or Topro (nuclei). Optimized confocal sections were done on Leica SPE or Zeiss710 microscopes for 3D reconstructions. The number of crystal cells, the volume (in μm^3^) of plasmatocytes and the volume (in μm^3^) of each anterior lobe, were measured using Volocity 3D Image Analysis software (PerkinElmer). Crystal cell index corresponds to the number of crystal cells/(primary lobe volume/10,000). Plasmatocyte index corresponds to the plasmatocyte volume/primary lobe volume. At least 15 anterior lobes were scored per genotype. Statistical analyses *t*-test (Mann–Whitney nonparametric test) were performed using GraphPad Prism 5 software.

### Intensity ratio for dfz3-RFP

The mean intensity of dfz3-RFP staining per cell was determined using Fiji software. To calculate the intensity ratio of dfz3-RFP in PSC cells, the mean intensity of dfz3-RFP staining for five randomly selected PSC cells was divided by the mean intensity of dfz3-RFP staining for five randomly selected MZ or CZ cells. Statistical analyses *t*-test (Mann–Whitney nonparametric test) were performed using GraphPad Prism 5 software.

### 3D reconstruction movies

LifeactinGFP was expressed in WT and *robo KD* PSC cells. Optimized *z* stacks where performed on Zeiss 710 confocal microscope. PSCs 3D reconstruction and rotation were performed using 3D viewer plugin of Fiji software.

### Data availability

The authors declare that the data supporting the findings of this study are available within the article and its Supplementary Figs.

## Additional information

**How to cite this article:** Morin-Poulard, I. *et al*. Vascular control of the *Drosophila* haematopoietic microenvironment by Slit/Robo signalling. *Nat. Commun.* 7:11634 doi: 10.1038/ncomms11634 (2016).

## Supplementary Material

Supplementary Information Supplementary Figures 1-8

Supplementary Movie 1 3D reconstruction of LGs expressing lifeactinGFP (green) in the wild type PSC.

Supplementary Movie 2 3D reconstruction of LGs expressing lifeactinGFP (green) in the robo KD PSC.
