# Supplementary material for: Vascular control of the Drosophila haematopoietic microenvironment by Slit/Robo signalling
Source: Nat Commun. 2016 May 19;7:11634. doi: 10.1038/ncomms11634 (PMC4874035; doi:10.1038/ncomms11634)
Supplement: Supplementary Information — Supplementary Figures 1-8 [file ncomms11634-s1.pdf]

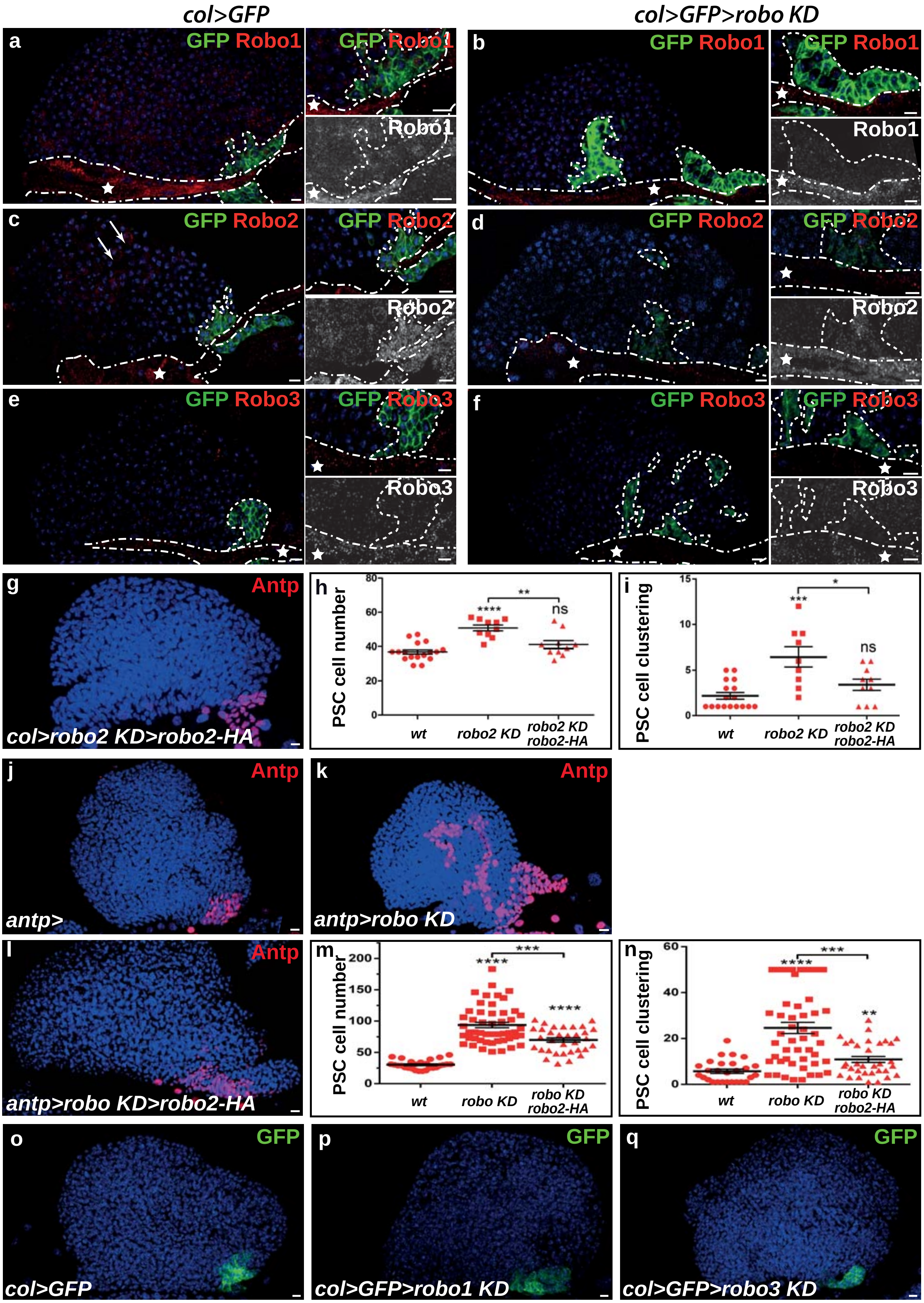

Supplementary Figure 1

Robo 1, 2 and 3 are expressed in the LG and are are required to control PSC morphology .

(a-f) PSC cells express GFP (green) under the PSC col driver in wt (a, c, e) and in robo KD (b, d, f). LGs (large left panels) and enlarged views of PSCs (small right panels) stained by Robo1 (red upper panels and white lower panels in a and b), Robo2 (red upper panels and white lower panels in c and d) and Robo3 (red upper panels and white lower panels in e and f). Arrows indicate crystal cells and a star indicates the cardiac tube. (g) Antp (red) labels PSC cells in col>robo2 KD>robo2-HA. (h, i) Quantification of PSC cell number (h) and PSC cell clustering (i). (j, k) Antp (red) labels PSC cells in wt (j) and in antp>robo KD (k). (l) Antp (red) labels PSC cells in antp>robo KD>robo2-HA. (m,n) Quantification of PSC cell number (m) and PSC cell clustering (n). (o-q) PSC cells express GFP (green) under the PSC col driver in wt (o), in robo1 KD (p) and in robo3 KD (q). Statistical analysis t test (Mann–Whitney nonparametric test) was performed using GraphPad Prism 5 software. Nuclei are labelled with DAPI (blue). Scale bars: 10µm

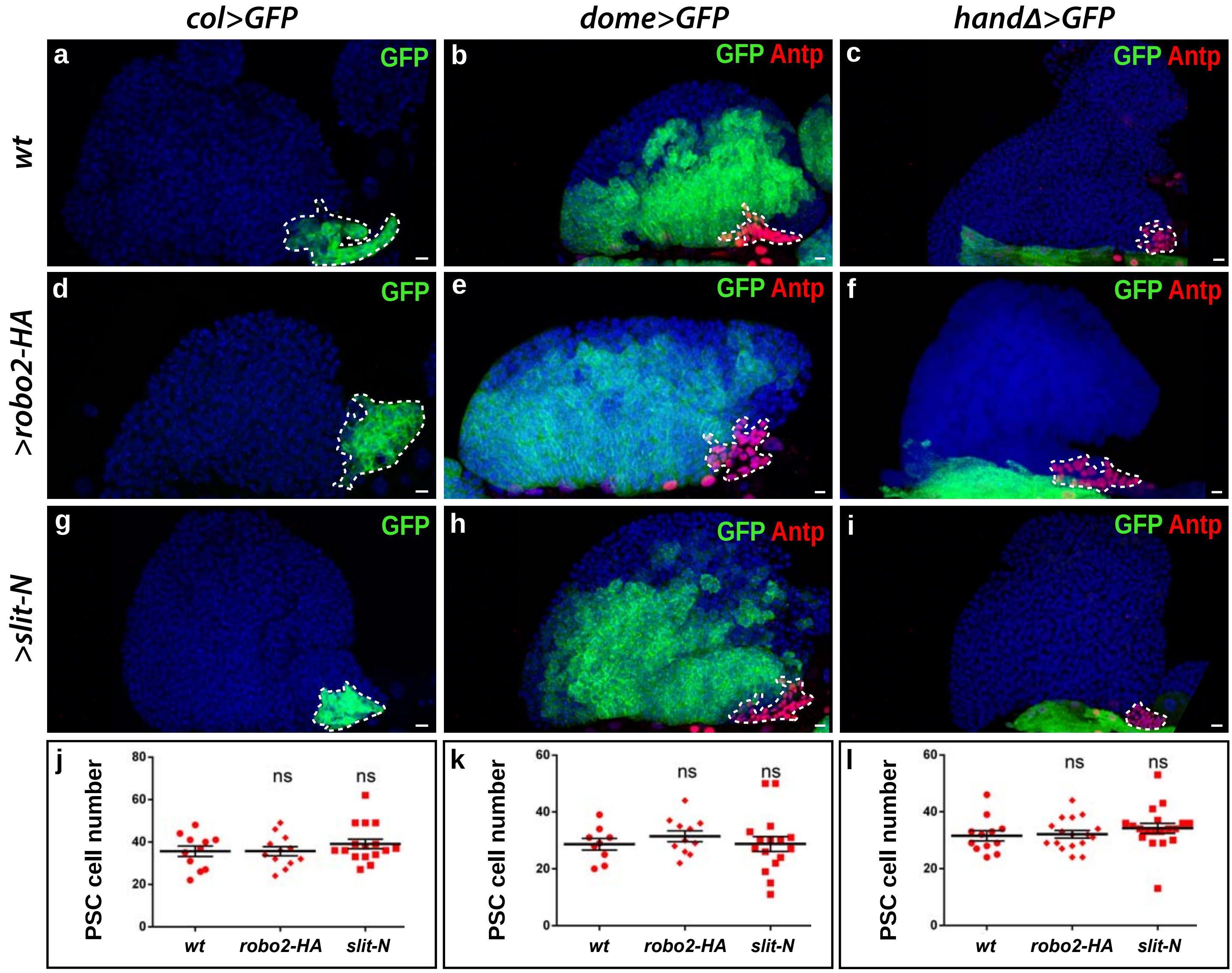

Supplementary Figure 2

Ectopic expression of robo2 or slit in the LG does not affect PSC morphology.

(a, d, g) PSC cells express GFP (green) under the PSC col driver in wt (a) and in conditions of robo2-HA (d) or slit-N (g) over/ectopic expression. (b, e, h) Antp (red) labels PSC cells, GFP (dome>GFP, green) labels MZ cells in control (b) and in conditions of robo2-HA (e) or slit-N (h) ectopic/overexpression. (c, f, i) Antp (red) labels PSC cells, GFP (handΔ>GFP, green) labels CT cells in control (c) and in conditions of robo2-HA (f) or slit-N (i) ectopic/overexpression. (j-l) Quantification of PSC cell number when col (j), domeless (dome) (k) or handΔ(l) drivers are used. Statistical analysis t test (Mann–Whitney nonparametric test) was performed using GraphPad Prism 5 software. Nuclei are labelled with Topro (blue) (a-i). Scale bars: 10μm.

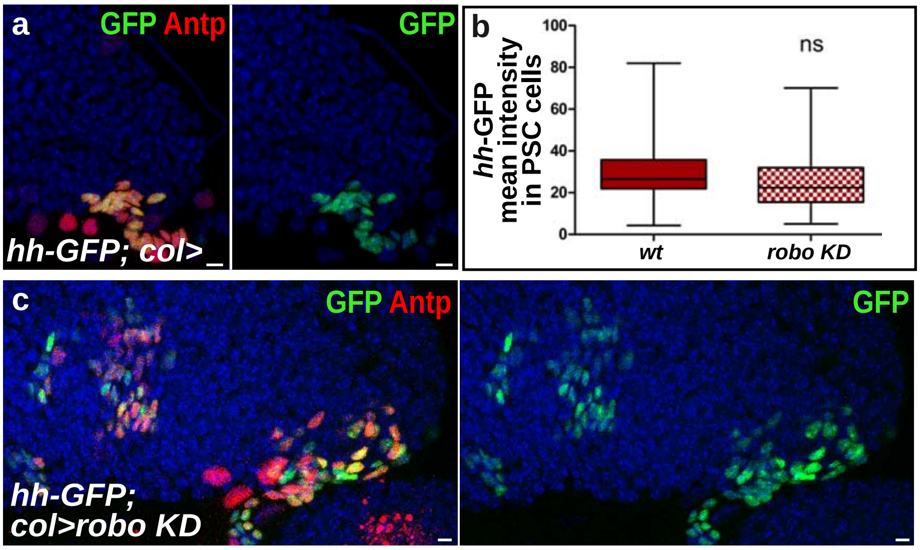

Supplementary Figure 3

Robo receptors are not required for Hh expression in the PSC .

(a-c) Hedgehog-GFP (hh-GFP, green) labels PSC cells in control (a) and in robo KD LGs (c). Antp (red, left panels) labels PSC cells (a, c). (b) Quantification of Hh-GFP mean intensity in PSC cells. Statistical analysis t test (Mann–Whitney nonparametric test) was performed using GraphPad Prism 5 software.

Nuclei are labelled with Topro (blue) . Scale bars: 10µm.

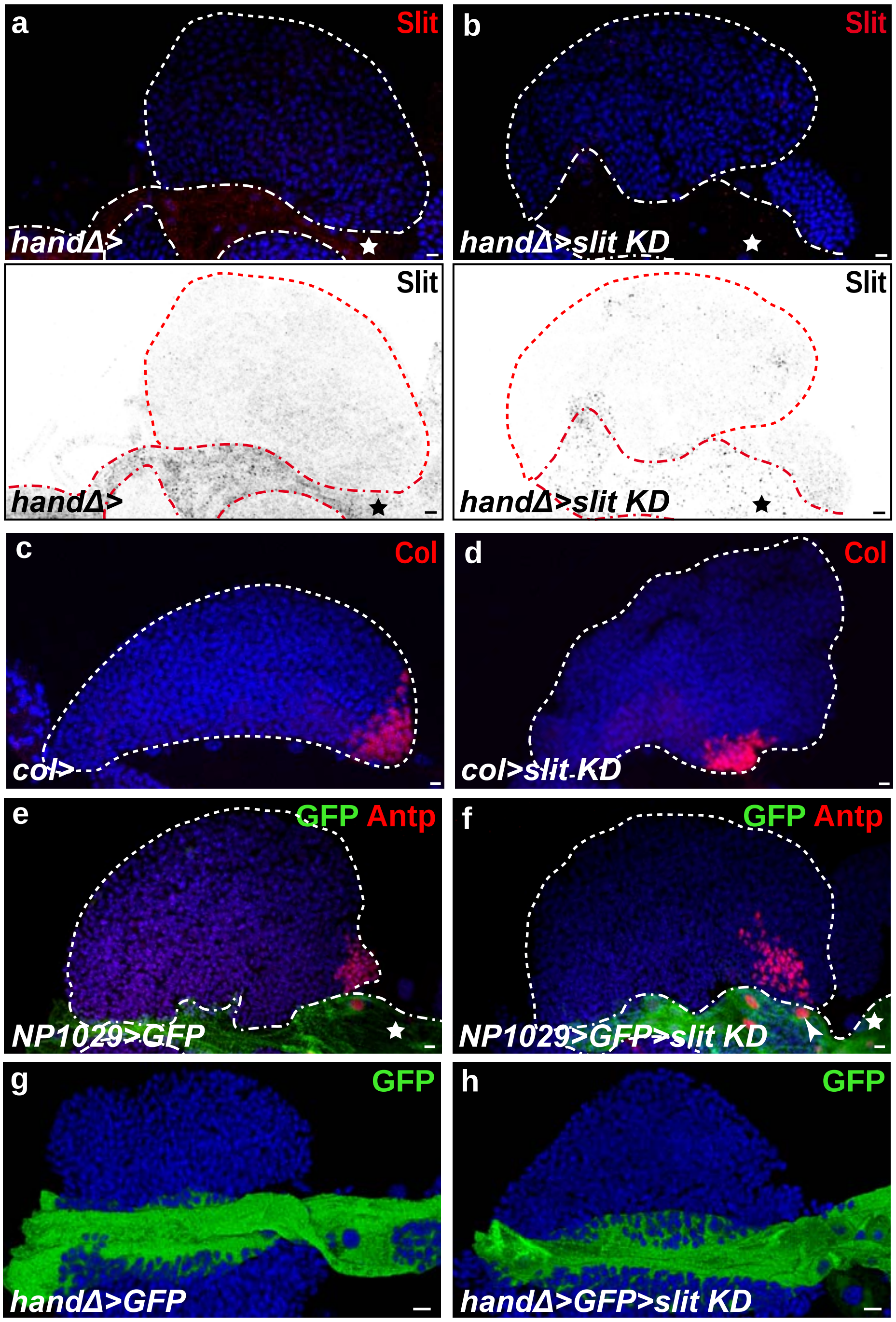

Supplementary Figure 4

slit dsRNA treatment strongly reduces the amount of Slit in the cardiac tube (CT) and does not affect CT morphology.

(a) hand Δ > and (b) hand Δ >slit KD LGs stained for Slit (red in upper panels ; black in lower panels). Dashed lines delineate the CT (*) and the LG. (c, d) Col (red) labels the PSC in control (c) and in LG where Slit is decreased in PSC cells (col>slit KD) (d). No defect in PSC morphology is observed in col>slit KD. (e, f) Antp (red) labels PSC cells in control (NP1029>GFP)(e) and in slit KD mutant(NP1029>GFP>slit KD) (f). Antp (red) labels also cardiac cells (arrowhead in f). (g, h) (GFP) labels the CT in control (hand Δ >GFP) (g) and in slit KD (hand Δ >GFP>slit KD) (h). 3D reconstructions of the CT, indicate that no defect in CT morphology is observed in slit KD.
None of the two CT drivers, Hand Δ (g) or NP1029 (e) are expressed in the LG. Nuclei are labelled with DAPI (blue).
Scale bars: 10µm.

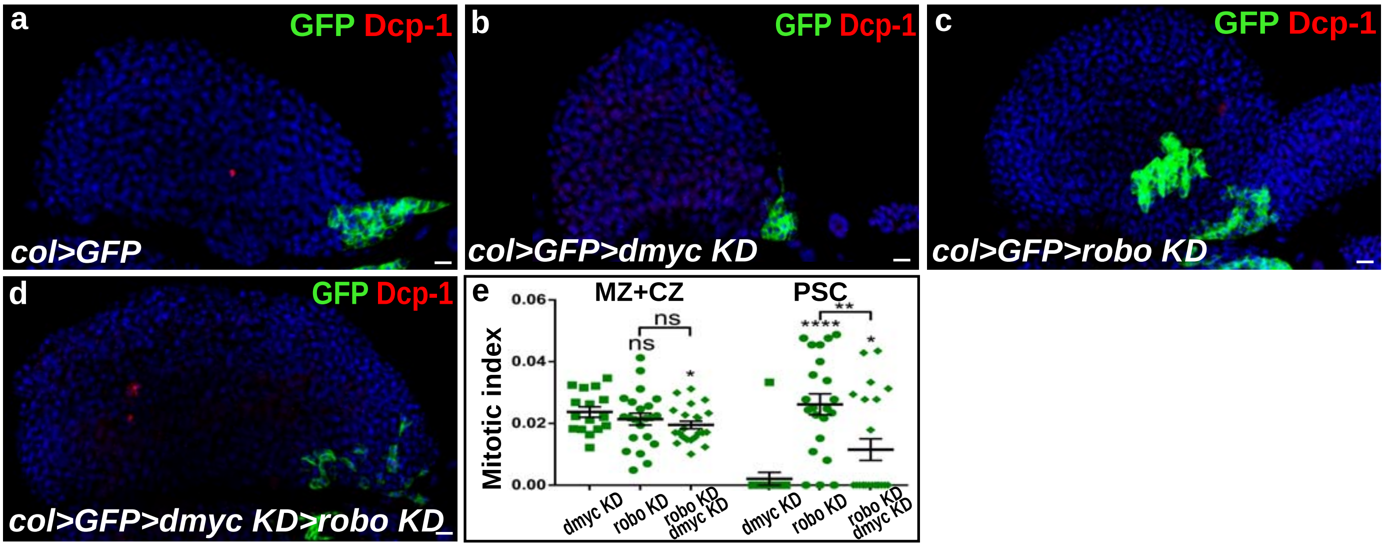

Supplementary Figure 5

Robo receptors in the PSC control PSC cell proliferation through dmyc.

(a-d) Dcp-1 staining (apoptosis, red) in the presence or absence of robos and dmyc in PSC cells (green).

(e) Mitotic index in the PSC and (MZ+CZ) in col>dmyc KD, col>robo KD and col>robo KD>dmyc KD. Statistical analysis t test (Mann–Whitney nonparametric test) was performed using GraphPad Prism 5 software.

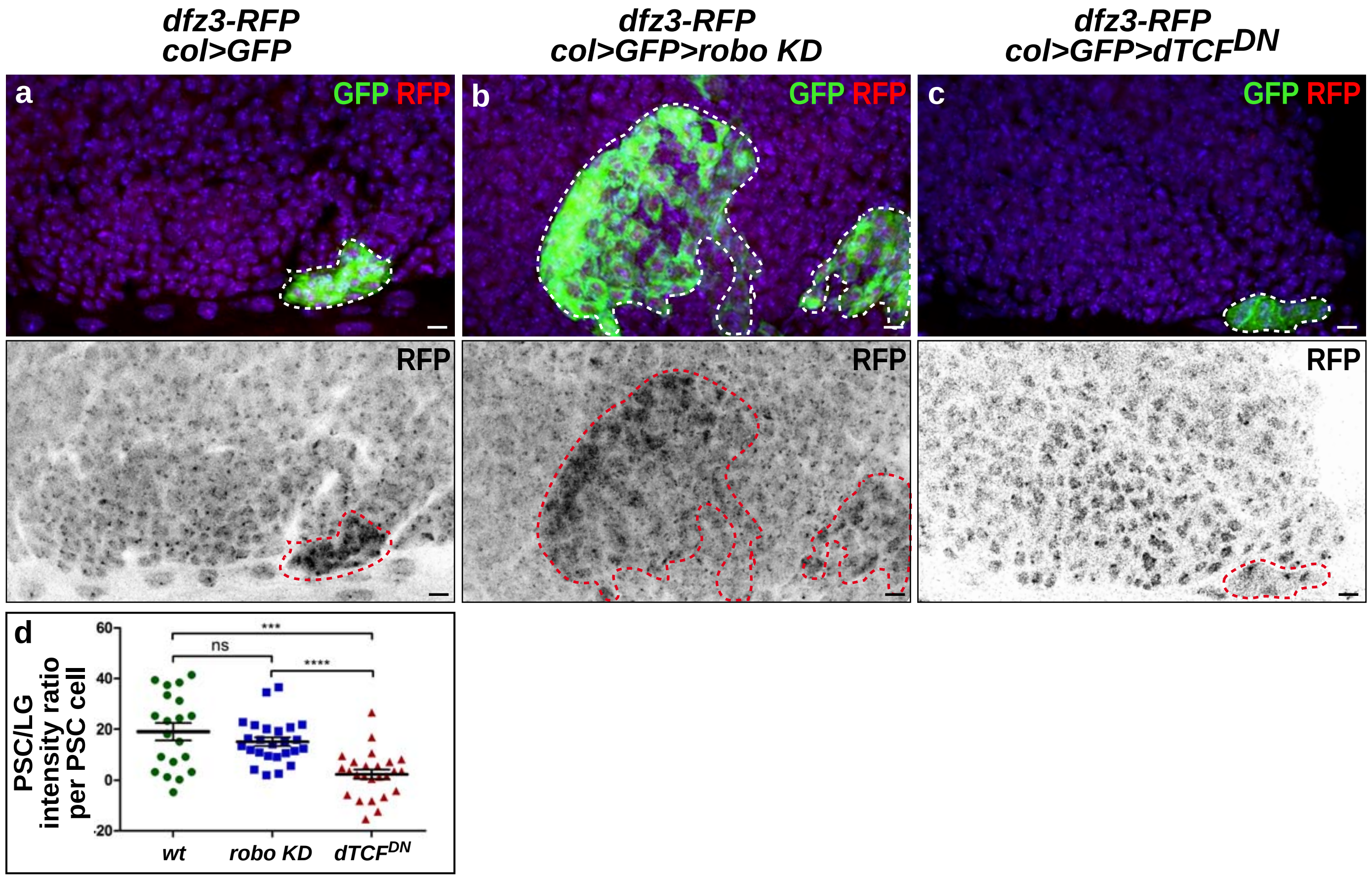

Supplementary Figure 6

Activity of the Wnt/Wg pathway is unaffected in roboKD PSC.

(a-c) frizzled 3 (dfz3-RFP,red in upper panels; black in lower panels) and GFP (upper panels) under the control of the col driver (col>GFP) in wt PSC (a), in robo KD PSCs (b) and in a PSC expressing a Dominant Negative form of dTCF (dTCFDN) (c). Whereas a weaker than normal RFP staining is observed in the PSC cells when the Wnt/Wg pathway is inactivated (c) no change is observed in robo KD (b) compared to wt PSC (a). Dashed line visualizes the PSC. (d) The intensity of RFP per PSC cell relative to other LG cells is given. Statistical analysis t test (Mann–Whitney nonparametric test) was performed using GraphPad Prism 5 software. Nuclei are labelled with DAPI (blue) (a-c). Scale bars: 10μm.

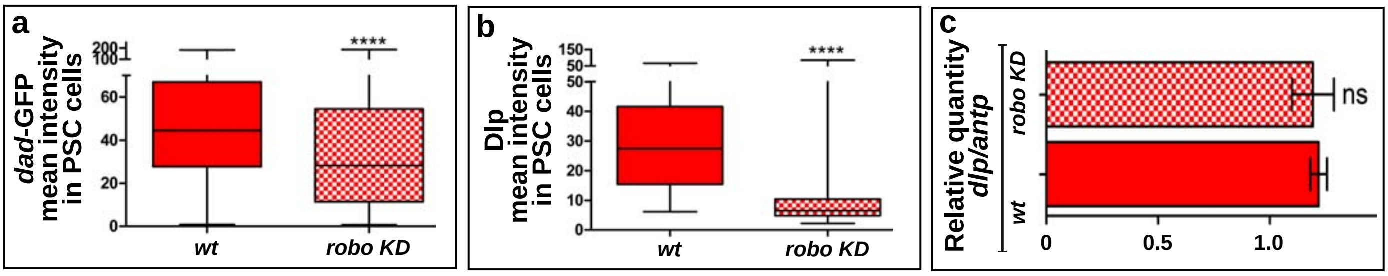

Supplementary Figure 7

Measurement of dad-GFP and Dlp intensity in robo KD mutant.

(a, b) dad-GFP (a) and Dlp (b) mean intensity in PSC cells in wt and in robo KD. (c) Quantitative RT-PCR of dlp relative to antp in wt and robo KD dissected LGs. Statistical analysis t test (Mann–Whitney nonparametric test) was performed using GraphPad Prism 5 software.

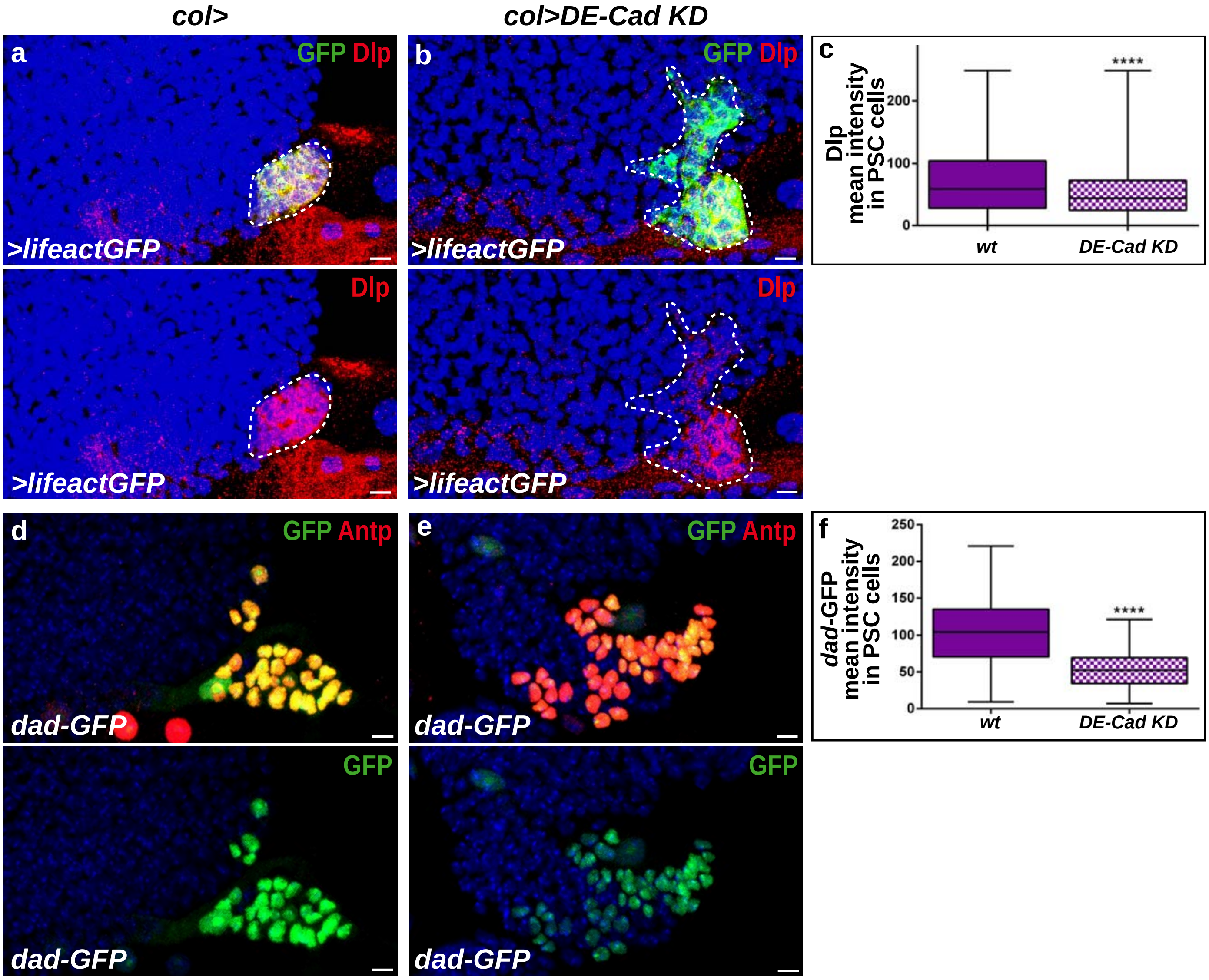

Supplementary Figure 8

DE-Cadherin controls Dlp and BMP/Dpp activity in the PSC.

(a-b) the PSC is visualized by lifeactGFP (green) in control (a) and DE-Cad KD (b) and Dlp expression is in red. Dashed line visualizes the PSC. (c) Quantification of Dlp means intensity in PSC cells. (d-e)Antp (red) labels the PSC in control (d) and in DE-Cad KD (e), dad-GFP is in green. (f) Quantification of dad-GFP mean intensity in PSC cells. Decreasing DE-Cadherin in the PSC leads to the decrease of both Dlp (b, c) and dad-GFP (e, f) expression in PSC cells. Statistical analyseis t test (Mann–Whitney nonparametric test) was performed using GraphPad Prism 5 software. Nuclei are labelled with Topro (blue). Scale bars: 10µm.
